# Modified streptavidin–biotin based lateral flow test strip for rapid detection of SARS-CoV-2 S1 antigen in saliva samples

**DOI:** 10.1038/s41598-024-57230-8

**Published:** 2024-03-27

**Authors:** Manal Kamel, Shimaa Atta, Sara Maher, Hesham Abd Elaziz, Zeinab Demerdash

**Affiliations:** 1 Immunology Department, Theodor Bilharz Research Institute, Giza, Egypt; 2 Surgery Department, Theodor Bilharz Research Institute, Giza, Egypt

**Keywords:** Streptavidin, Biotin, Nanobodies, SARS-CoV-2 S1, Saliva, Rapid test, Biotechnology, Diseases

## Abstract

Compared to other infectious diseases, for which LFT development can take years, SARS-CoV-2 antigen LFTS were developed and deployed within months. LFTS for antigen detection were adopted on an unprecedented scale during the COVID-19 pandemic, but many of them lack the sensitivity especially for samples with low viral load. In our previous work, we developed an enhanced signal strip for detection of SARS CoV-2 SI antigens in saliva. Here we introduce some modification to improve the sensitivity, and specificity, and to lower the cost of the strip, by using biotin streptavidin (BS) system. In the modified BS strip, gold-streptavidin and biotinylated Nanobodies (Nbs) against S1 antigen were externally mixed with the tested samples (saliva or nasopharyngeal swab) before their application on the sample pad of the test strip containing angiotensin converting enzyme (ACE-2), as the capturing probe. The study included 320 individuals, with 180 being positively confirmed by RT-PCR and 140 confirmed negative, as well as, 45 health care workers, who were responsible for screening and handling of surgical cases in General Surgery Department and COVID clinic of TBRI. Our results proved that modified BS strip improved the overall sensitivity and specificity of S1antigen detection in saliva samples (95.21% and 99.29% respectively) compared to our previously developed enhanced LFTS (91.66% and 98.57% respectively). Also, the sensitivity of cases with Ct ≤ 30, Ct $\le$ 35, and Ct $\le$ 40 using the modified BS strip showed higher values (98.54%, 95.38%, and 88.89% respectively), compared to the corresponding results of our previously developed enhanced LFTS (95.86%, 92.31%, and 82.22% respectively). There were no cross-reactions with either Middle East respiratory syndrome corona virus MERS-CoV or SARS-CoV antigens. Furthermore, we found that the lower viral detection limit (LVD) of BS strip was obviously lower than our previous LVD limit of the enhanced LFTS (0.2 × 10^4^ copies/ml vs. 0.4 × 10^4^ copies/ml, respectively). Our developed BS strip showed that saliva samples gave better results than nasopharyngeal swabs of the same patients. The fact of using smaller amounts of Nbs, and ACE2, as well as the dispensing off of conjugate pad when applying BS strip modifications, justified the expected reduction in the costs of the strip. The implementation of BS strips on saliva samples of 45 health co-workers, who were tested 4 and 6 days after exposure to infection, showed an increase in the sensitivity, starting from the 4th day and reaching its highest level on the 6th day in both high risk and paramedic groups (90.9%, and 80.0%, respectively). This study provides evidence that employment of the modified BS system could increase the sensitivity of the strips, lower their cost, and render them an effective screening tool for early detection of the virus in saliva of suspected Covid-19 patients.

## Introduction

The world health organization (WHO) declared that the corona virus disease 2019 (COVID-19) became a global pandemic after its rapid spread all over the world since its early recognition in December 2019, in Wuhan, China^1^.

The spread of COVID-19 can be controlled by accurate diagnosis bringing about early treatment of infected cases^2^. Although the RT-PCR detection of viral RNA in nasopharyngeal swabs is the gold standard method for diagnosis, the current clinical experience implies that it has many drawbacks^3^.

However, concerns remain surrounding the performance of antigen tests due to their low detection sensitivity, especially in samples with low viral load. Recent studies showed that antigen tests demonstrated a wide range of sensitivity from 16.7 to 85%^4^. Hence, in our previous work we tried to employ Nanobodies to replace mAbs as a more sensitive detector of SARS S1 protein. Due to their small size, simple structure, high antigen binding affinity, and remarkable stability in extreme conditions, using nanobodies overcame several of the limitations of conventional monoclonal antibodies and gave promising results in ELISA with overall sensitivity and specificity of 88.7% and 100%, respectively^5^. Subsequently, we developed an enhanced LFTS using dual gold conjugation of ACE-2 and Nbs that resulted in 91.66% sensitivity and 97.57% specificity^6^.

Streptavidin is a basic glycoprotein composed of four identical subunits; each binds to biotin with high affinity (Kd ~ 10–15 M)^7^, exhibiting low level of nonspecific binding. Compared to other covalent and non-covalent bonds, the avidin–biotin system provides many advantages such as; amplification of weak signals which enables the use of highly diluted primary antibodies^8^, as well as, its high affinity interactions, which are stable against proteolytic enzymes, pH, temperature, and denaturing reagents^9^.

In the current study, we tried to improve sensitivity and specificity of our previously developed LFTS, using the biotin–streptavidin system, through the employment of gold–streptavidin and biotinylated anti-S1 recombinant Nbs as detector probes, and ACE-2 protein as a capture probe, for detection of SARS-CoV-2 spike protein (S1) in saliva samples. All results of BS LFTS on saliva samples were compared with that of nasopharyngeal swabs of the same patients.

## Materials and methods

### Ethical approval

This study was approved by the Research Ethics Committee (REC) at Theodor Bilharz Research Institute (TBRI) (#PT 623, 2021). The human subjects in this study were enrolled according to REC-TBRI’s ethical standards and the 1964 Helsinki Declaration. A signed consent form was obtained from each participant before sample collection.

## Materials and equipment

Bovine serum albumin (BSA) and sucrose (C12H22O11) skimmed milk were purchased from Sigma Aldrich, USA. Recombinant human coronavirus SARS-CoV-2 spike glycoprotein S1 (ab 288546), Recombinant angiotensin-converting enzyme 2 (ACE2) (ab151852), Anti llama antibody (ab112784), Streptavidin-gold (ab186864), Antibody-Biotin conjugation kit (ab201795) that was previously available in our lab. All from Abcam, Cambridge, UK. SARS-CoV recombinant protein (MBS569928), MERS CoV spike S1 (MBS434229) antigen from MyBioSource, California, US. SARS-CoV-2 S1 nanobodies (A13PACC00V003, AssayGenie, Dublin, Irland). A sample pad and absorption pad (cat no CFSP173000), high flow nitrocellulose membrane (NC) (cat no HF09002XSS), Merch Millipore (Darmstadt, Germany). UV–Vis-spectrophotometer (Thermofisher-USA). Manual dispenser (Nanomat 4-CAMAG-laborto), PH meter (Jenway 3510, UK), High-speed centrifuge (Eppendorf, 5430R, Germany). Ultrapure water used throughout was generated from a Millipore Milli-Q water purification system (Billerica, MA, USA). Gel documentation system (Gel Doc XR +) (Biorad, USA) & data analysis by “Image lab” software version 3.0 (https://www.bio-rad.com/en-eg/category/geldoc-go-gel-imaging-system?ID=O494SO15)_. JEOL JSM5200 Scanning Electron Microscope, Japan.

### Clinical samples

This study comprised both saliva and nasopharyngeal samples collected from 320 patients with COVID-19 symptoms (45% males and 55% females, aged 35–65 years old) 4–6 days after starting common symptoms (fever, cough, bone aces, diarrhea, headache, sore throat, skin rash, loss of taste or smell, difficulty in breathing, chest pain or pressure). In addition, 45 health care workers including 25 from high risk group (Clinicians, surgeons, radiologists and nurses) and 20 from paramedic group (technicians) were also tested. Samples were collected from COVID-19 outpatient clinic at TBRI (June 2021-April 2022), in accordance with relevant guidelines and precautions of WHO. At the outpatient clinic, screening for COVID 19 antigen was performed on nasopharyngeal swabs of all individuals included in the study using commercially available rapid antigen detection kit (Bright Sign, China). Nasopharyngeal swabs were diluted in specific solution provided in the kit, and the remaining part of the solution was preserved at -80 for future use by our developed strips. As mentioned in our previous paper (6), two ml of un-stimulated saliva were self-collected by patients included in this study, early in the morning. They were asked to wash their mouth with water then to spit repeatedly into sterile cups, which were securely closed and preserved at -80 till used. On application of our developed strip, saliva samples were thawed and used as such without dilution.

Patients were divided according to RT-PCR results into 180 confirmed positive and 140 symptomatic confirmed negative.

### RT-PCR testing of collected NP samples

RT-qPCR was performed for all collected nasopharyngeal swabs by the national reference lab using One-step Real Time RT-PCR Master Mixes kits (Thermo Fisher, Waltham, MA, USA) for SARS-CoV-2 typical N and ORF1ab target genes. The assay was performed according to the manufacturer’s protocol of the kit.

### Preparation of streptavidin-gold

Streptavidin-gold was shipped in TBS buffer, so, an extra centrifugation step at 9000 g was performed at first, then the supernatant was removed, and precipitate was dissolved in PBS, which is the same buffer to be used in all following steps of strip development.

### Biotin labeling of Nbs for conjugation to streptavidin–AuNPs

SARS-CoV-2 S1 Nbs were biotinylated using Antibody-Biotin conjugation kit. According to kit instructions, 1 µL of modifier reagent was mixed gently with 10 µL of Nbs (trying three different concentrations of Nbs; 5 µg, 10 µg, and 15µg diluted in PBS pH7.4, for optimization purpose). The blend was mixed with lyophilized Biotin Conjugation Mix vial, and left standing at RT in the dark for 20 min. Then, 1 µL of Quencher reagent was added and mixed gently. The conjugates were stored at 4 °C till used. Moreover, we compared the performance of stored biotinylated Nbs versus freshly prepared ones in LFTS.

### Preparation of sandwich based lateral flow test strips (LFTSs)

#### Principle

Biotin/streptavidin sandwich based LFTS was employed for detection of SARS-CoV-2 S1 antigen in saliva. At first, gold-streptavidin and biotinylated Nbs were externally mixed with the tested samples in sterile vials before their application on the sample pad to form a complex with ACE-2 on the test line. If the tested sample contained S1 antigen of SARS-CoV-2, it would bind to biotinylated Nbs and gold conjugated streptavidin. The complex would migrate to the membrane-bound ACE2 protein, at the test line, turning it red. In the meantime, the unbound conjugates would continue to flow forward to bind to anti-llama antibody on the control line, turning it red. Therefore, the LFTS was either positive or negative according to red color development on the test line. If no red color appeared on the control line, the test result was considered invalid, regardless of the color on the test line.

#### LFTS components

**Figure 1:**
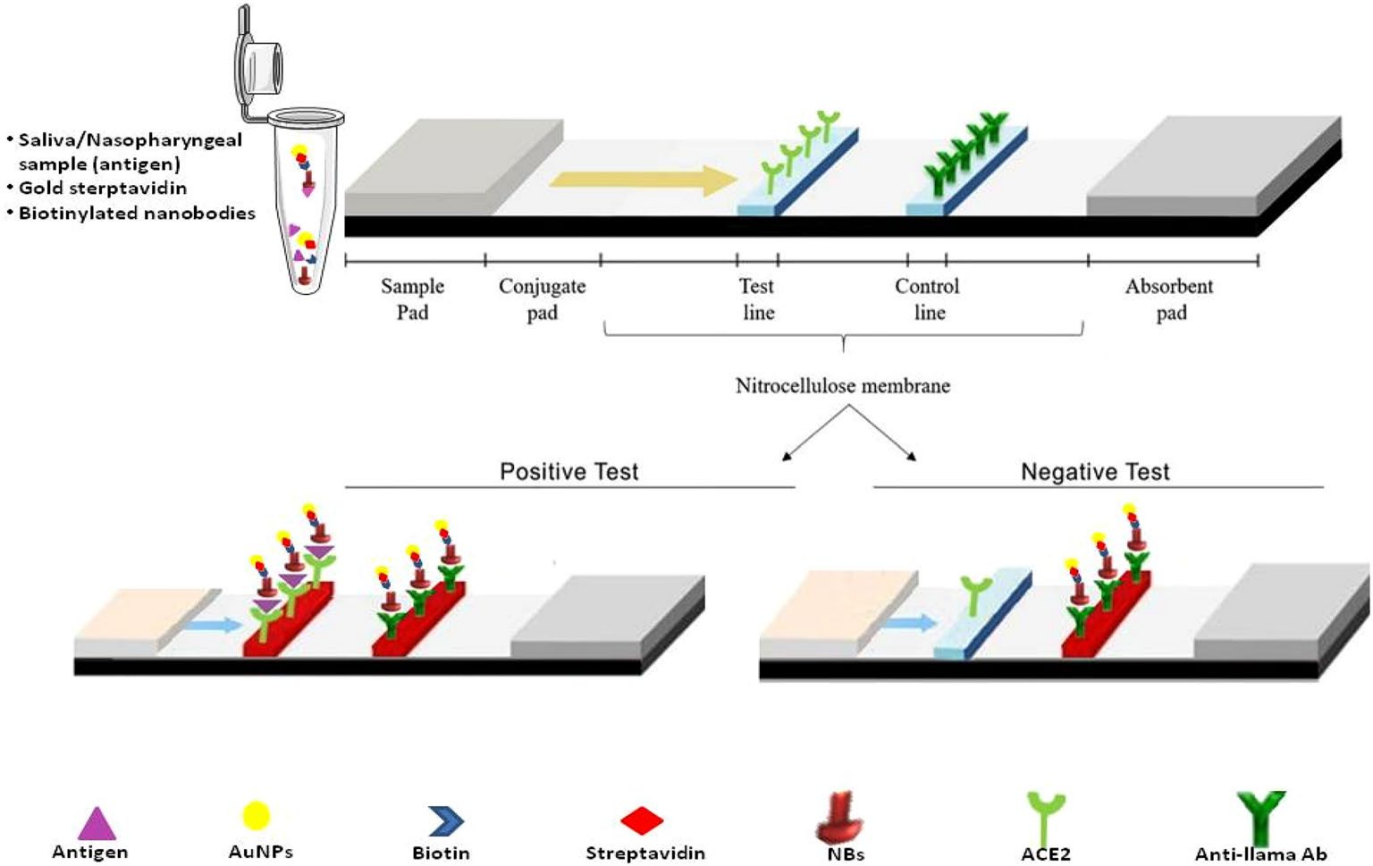
Diagrammatic representation of the principle of biotin-streptavidin (BS) sandwich LFTS for detection of SARS-CoV-2 S1 antigen in saliva samples or nasopharyngeal swabs.

The structure of BS strip is presented in Fig. 1. It is composed of three parts: a) sample pad (cat no CFSP173000), b) a high flow nitrocellulose membrane (NC) (Merch Millipore, Darmstadt, Germany, Cat no HF09002XSS), and c) an absorption pad (cat no CFSP173000).

### Optimization of LFTS parameters

Several parameters were optimized during the development of the LFTS including: Test line and control line preparation steps, nitrocellulose membrane (NC) blocking step, vial mixtures (gold-streptavidin, and biotinylated Nbs conjugate concentrations, sample volume).

#### Optimum concentrations of coating proteins at test & control lines

Different volumes of diluted test line coating protein; ACE2 (2, 5, 10 μl/strip) were tested. For control line protein (anti-llama IgG), 3 and 5 μl of 1 and 2 mg/ml per strip, were tested. Optimum concentrations were specified when a distinct red color of control and test lines were reached, following testing the same samples under the same conditions.

#### NC membrane blocking optimization

Different immersion times (10, 15 and 20 min) were tested using the blocking buffer of 50 mM boric acid buffer 0.3% (w/v) skimmed milk (pH 8.5).

#### Sample volume and condition

The vial-mixtures were tested several times with different concentrations of biotinylated-Nbs and gold-streptavidin as well as different volumes of the applied samples (saliva or nasopharyngeal swabs).

### Morphological characterization of the LFT strip

Atomic force microscopy (AFM) was performed by AFM instrument model of 5600Ls manufactured by Agilent Technology, USA. The analyses were performed in tapping mode in different sizes, using phase contrast and height modes. At least six images of different areas were obtained and the best representative images were selected. The images were processed with Agilent’s PicoView 1.5 imaging and analysis software package.

### Characterization parameters of LFTS Performance

Evaluation of LFTS performance to determine the efficiency and accuracy of the test was carried out through testing of the following characterization parameters:

#### Determination of lower viral detection limit, Cross reactivity, and stability conditions of the developed LFTS

- Determination of lower viral detection limit
  Lower viral detection limit (LVD) was obtained by using serial dilutions (2.7 × 105 till 0.2 × 104 copies/ml) of γ-radiated SARS-CoV-2 ( hcov-19/Egypt/NRC-03/2020-SARS-CoV2 Strain; GISAID accession#EPI-ISI-430819), that was kindly donated by Centre of Scientific Excellence for Influenza Virus, Environmental research Division, National Research Centre-Egypt*** .***
  - Testing for cross reactivity of developed lateral strips with related viruses:
    Cross reactivity was tested for by using two different corona-related spike antigens (SARS-CoV S1, and MERS S1) that were prepared in the sample buffer.
- Determination of the favorable stability conditions for storage of LFTSs
  The developed test strip was tested for its storage stability at different temperatures (4 °C, RT, and 37 °C), and at different periodic times (1, 2 and 4 months) to determine the best conditions for optimum effectiveness of LFTS.

#### Application of clinical samples on developed LFTS

10 µl of each sample (saliva or nasopharyngeal swabs) were applied to the developed strip and incubated at RT for 10 min. The color intensity was then evaluated to discriminate positive from negative samples. Color intensity were detected by Gel documentation system as a volume using Image Lab software.

### Statistical analysis methods

(BS) strip was evaluated using RT-qPCR test as a reference test based on the following accuracy measure: sensitivity, specificity, positive predictive value, negative predictive value and Cohen’s kappa statistic (κ).

Kappa value was estimated to determine the degree of agreement between the PCR test and other techniques utilized in this study. The level of agreement was measured according to the following scale (Landis and Koch, 1977)^10^:

**Table:** 

k-value	Agreement level
0	Poor
0.01–0.2	Slight
0.21–0.4	Fair
0.41–0.60	Moderate
0.61–0.80	Substantial
0.81–1	Almost perfect

Statistical analyses were performed using the analytical software package (IBM-SPSS) version 23 (https://www.ibm.com/support/pages/downloading-ibm-spss-statistics-23). The receiver operating characteristic curve (ROC) was built to test characteristics of both BS- and enhanced-strip assays.

## Results

### Biotin labeled Nbs for conjugation to streptavidin–AuNPs

The best results were obtained by using 5 µg of Nbs (1mg/ml), this concentration showed better results on testing its reactivity with gold conjugated streptavidin. Furthermore, there was no change in results on application of stored or freshly prepared conjugate; both showed efficient results when used in BS strip.

### Standardization of the sandwich-based biotin avidin lateral flow test strip

**Figure 2:**
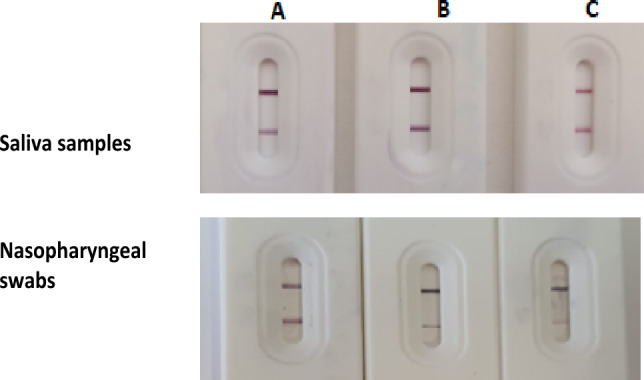
Optimization for BS based upon the immersion time for the same test sample (**A**) 20 min. immersion time (**B**) 15 min immersion time, (**C**) 10 min. immersion time. (**B**) showed the most identified control and test line for saliva while (**A**) showed the most identified control and test line for nasopharyngeal swabs (strongest red color).

Following numerous optimization trials, the optimal conditions were determined to be the parameters listed in Table 1 and visualized in Fig. 2.

**Table 1:** Optimization conditions for development of BS strips.

Sample volume (saliva/nasopharyngeal)	Test line	Control line	NC
In test tube mix of (a) 4 μl biotin-Nbs (b) 5 μl of gold-streptavidin (c) 5 μl of saliva sample as such or diluted nasopharyngeal swab	5μl of ACE-2 dil 1:7 in antibody buffer: 0.01M PBS, pH7.2 and 0.05 Tween 20	2 μl of Anti-Llama IgG 1mg/ml	15 min. in blocking buffer with 50mM boric acid buffer 0.3% (w/v) skim milk (pH 8.5)

### Fabrication of LFTSs

Following the adjustment of the optimization conditions, LFTSs were assembled by dispensing the test line and control line components (as listed in Table 1) on the NC membrane. The interval between the two lines was 4 mm. Then, the NC membrane was blocked by blocking buffer and left to dry at RT for 1 h. The absorbent pad was attached to the opposite side of the NC membrane. The complete assembled strip measured 4 × 60 mm. The LFTSs were stored in a sealed bag at RT until used.

### Morphological characterization of the LFT strip

**Figure 3:**
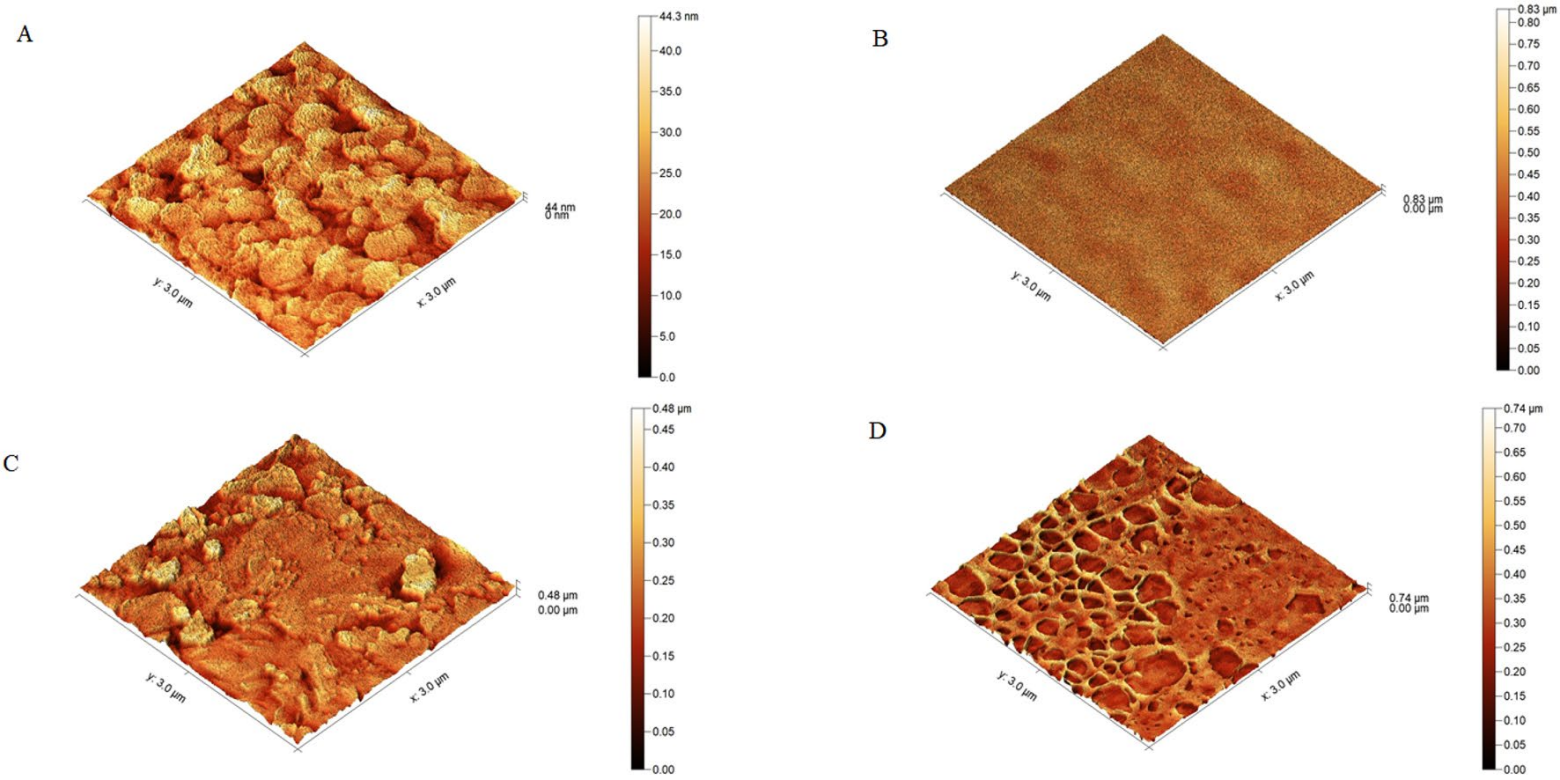
AFM image (**A**) test line with capture conjugate (**B**) sample pad (**C**) test line with sandwich complex, (**D**) nitrocellulose membrane.

AFM images, as shown in Fig. 3 represent the porous morphology for the NC membrane as well as the smooth structure of the sample pad sample used in the construction of the LFTS. Furthermore, the test line morphology before and after the formation of the immune complex with S1 antigen. The test line containing the capture probe (ACE-2) showed a structure of 44 nm while an increase in the height was observed following the formation of the immune complex (0.48 μm) represented as a globular structure, confirming the efficient formation of the immunocomplex with S1 antigen at the positive test line (ACE2 + biotinylated nanobodies-gold streptavidin S1 complex).

### Characterization parameters of LFTS Performance

**Figure 4:**
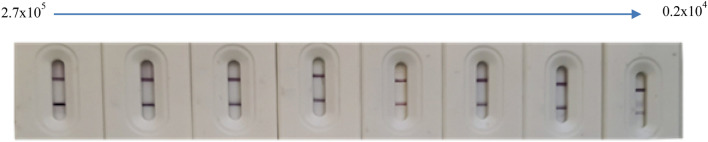
Determination of the lower viral detection limit (LVD) for BS-LFTS by using serial dilutions of inactivated SARS-CoV-2 starting from 2.7 × 10^5^.

**Figure 5:**
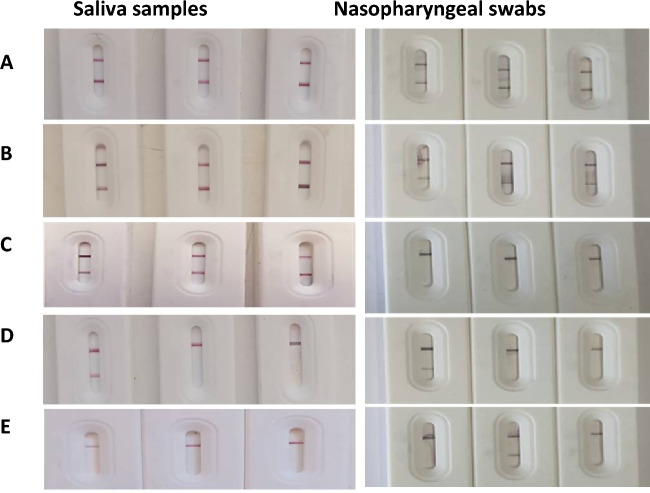
Stability testing for three positive saliva and Nasopharyngeal samples using BS-strips under different conditions. (A) before storage, (**B**) storing for 2 months at RT, (**C**) storing for 4 months at RT, (**D**) storing for 1 month at 4 °C, (**E**) storing for 1 month at 37 °C. Compared to the before storage strips, best storage conditions was observed for strips stored at RT up to 4 months with saliva samples (**C**).

- No cross reactivity was detected with the two different corona-related spike antigens (SARS-CoV S1, and MERS S1).
- The lower viral detection limit (LVD) was 0.2 × 104) as shown in (Fig. 4).
- The favorable stability conditions, allowing for accurate and reliable results of LFTS, were when stored at RT for up to 4 months (Fig. 5).

### Application of clinical samples on developed LFIS and statistical analysis of the results

In our study we used RT-PCR as a reference test to confirm COVID-19 positive cases, and the viral load values (Cycle thresholds), as a measurement for the disease intensity.

### SARS-CoV-2 S1 antigen detection by BS strip in saliva and nasopharyngeal swabs using BS strip

#### Accuracy measures

Sensitivity, Specificity, and Diagnostic efficacy of the BS strips in both saliva samples and nasopharyngeal swabs are listed in Table 2. The highest diagnostic efficacy percent (94.75%) was achieved with the use of saliva compared to 91.88% with nasopharyngeal swabs in BS strip.

**Table 2:** Accuracy measures; sensitivity, specificity, and diagnostic efficacy of the BS strips applied using both saliva samples and nasopharyngeal swabs.

	Saliva (%)	Nasopharyngeal (%)
Sensitivity	95.21	91.11
Specificity	99.29	98.57
Diagnostic efficacy	96.56	94.38

Table 3 showed that for most Ct values of tested saliva samples, kappa statistic was always greater than 0.8, reflecting perfect agreement level of BS strip results with RT-PCR. According to Ct values, the highest sensitivity was found at Ct $\le$ 30 by using BS with saliva.

**Table 3:** Accuracy measures of the BS strip assay categorized according to Ct values.

		Sensitivity (%)	Specificity (%)	PPV (%)	NPV (%)	Diagnostic efficacy (%)	Kappa
In saliva	Overall Ct	95.21	99.29	99.42	93.29	96.56	0.931
	at Ct $\le$ 30	98.54	99.29	98.55	98.58	98.57	0.968
	at Ct $\le$ 35	95.38	99.29	98.41	97.89	98.05	0.955
	at Ct $\le$ 40	88.89	99.29	97.56	96.53	96.76	0.909
In nasopharyngeal	Overall Ct	91.11	98.57	98.80	89.61	94.38	0.887
	at Ct $\le$ 30	94.29	98.57	97.06	97.18	97.14	0.935
	at Ct $\le$ 35	92.31	98.57	96.77	96.50	96.59	0.92
	at Ct $\le$ 40	84.44	98.57	95.00	95.17	95.14	0.863

#### ROC curve

**Figure 6:**
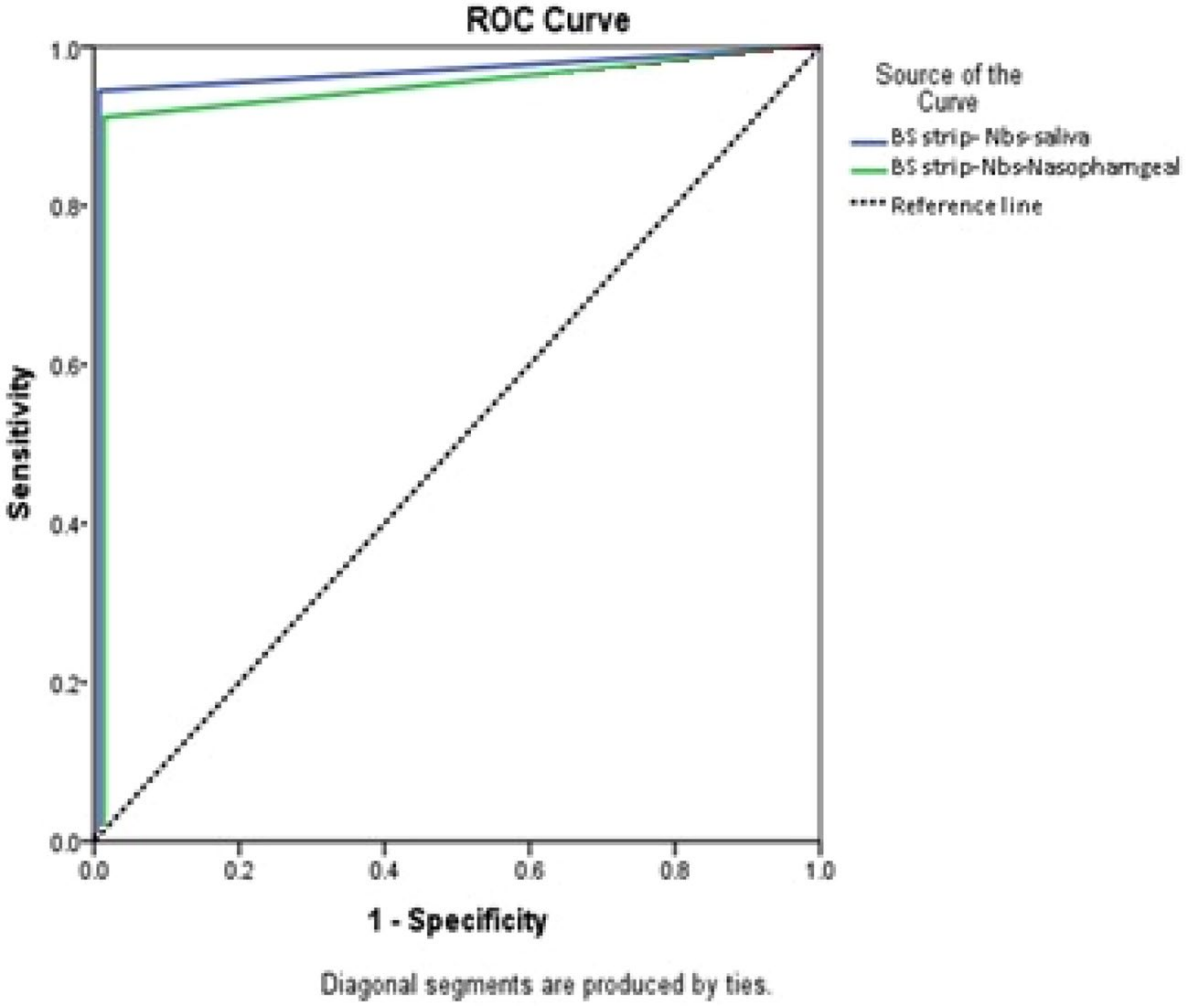
Receiver operating characteristic (ROC) curve of BS strip assay in saliva samples and nasopharyngeal swabs, using RT-PCR as a reference test. AUC, area under curve.

According to ROC curve analysis, BS-strip assay showed high AUC values in both saliva as well as nasopharyngeal swabs. In addition, AUC in saliva samples was higher than that in nasopharyngeal swabs (Fig. 6).

#### Color intensity readings of the BS strips test line using gel documentation system

**Figure 7:**
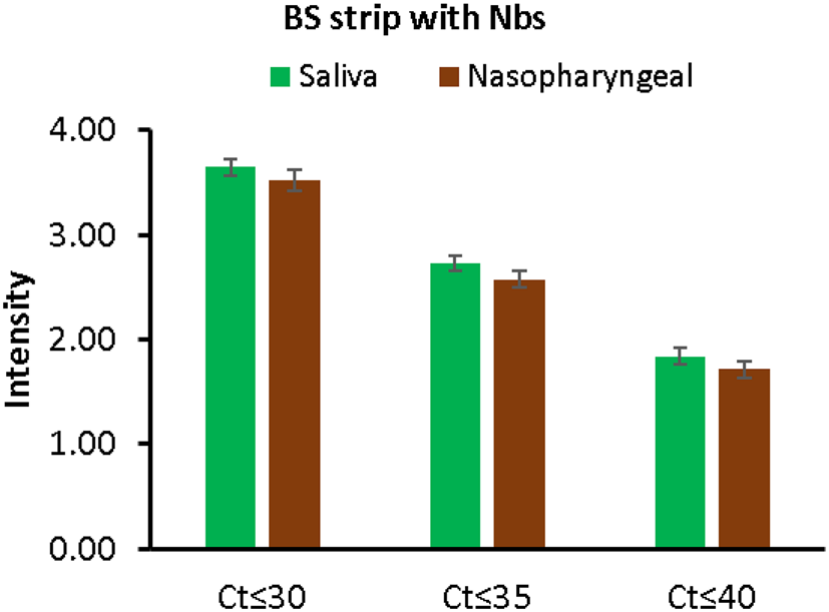
Color intensity of the BS strip’ test line for detection of S1 antigen in saliva and nasopharyngeal swabs with different Ct values (**A**). Bars represent mean ± standard error of mean.

Test line color intensity of the developed test strip reflects the SARS-CoV-2 S1 antigen concentration in the tested samples. The intensity of the BS strip test line for positive cases with different Ct values applied in saliva samples and nasopharyngeal swabs are displayed in Fig. 7. In saliva samples, the intensity using BS strip was significantly high at all Ct values. In nasopharyngeal swabs, there was significant decrease in the intensities in the cases with Ct ≤ 30 and 35 only.

**Table:** 

	AUC	SE	*P*-value	95%CI	
				Lower limit	Upper limit
Saliva	0.97	0.011	0.000	0.947	0.99
Nasopharyngeal	0.95	0.014	0.000	0.921	0.975

#### Correlation of color intensity between saliva and nasopharyngeal samples

**Figure 8:**
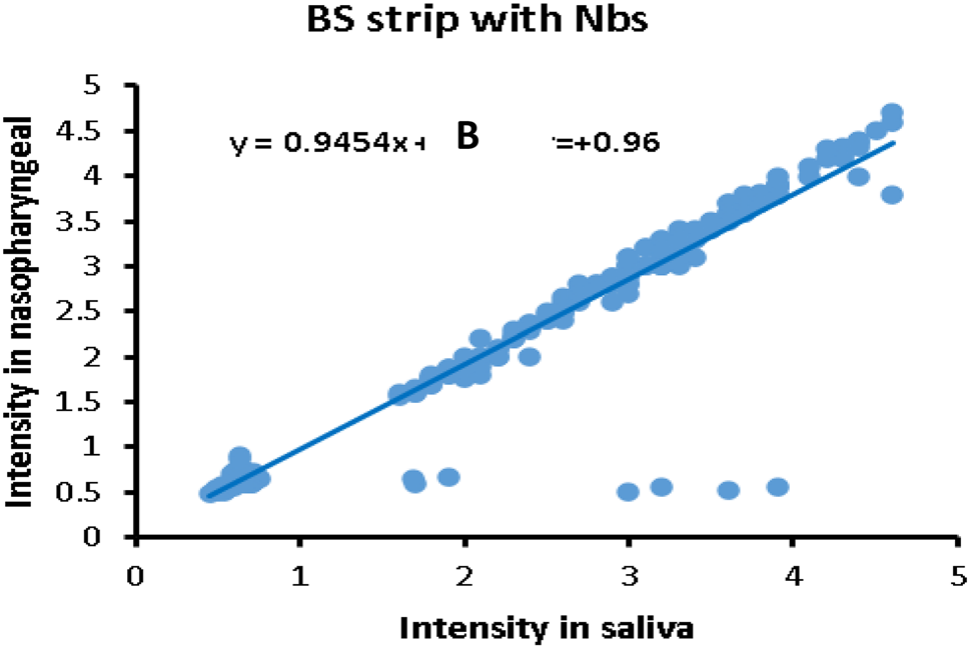
The relationship between color intensity of BS strip’ test line in saliva samples and nasopharyngeal swabs using BS strip (**A**). r: represent Pearson’s correlation coefficient between intensity in saliva and nasopharyngeal.

The fitting relationships of color intensity of BS strip on application in saliva and nasopharyngeal swaps are displayed in Fig. 8. We found a strong correlation of color intensity between saliva and nasopharyngeal samples (r = + 0, 96), indicating that saliva samples are a better replacement for the discomforting nasopharyngeal swabs.

**Figure 9:**
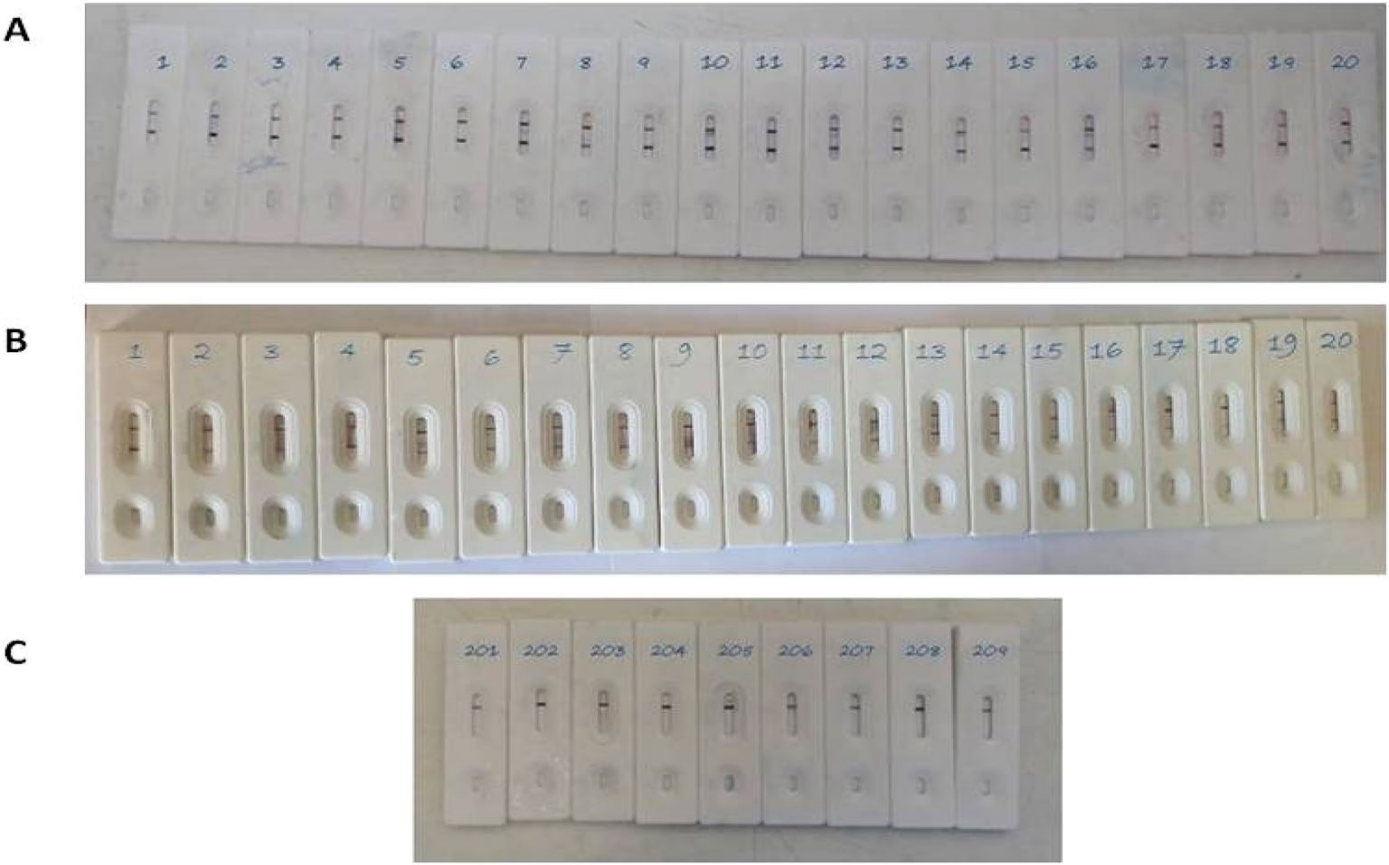
Application of some confirmed RT-PCR positive saliva samples (**A**), nasopharyngeal swabs (**B**) and negative saliva samples (**C**) on the developed LFTS showing variable color intensities of test line using BS strip.

Results of our developed BS strip with saliva samples of confirmed RT-PCR CoV-19 positive and negative cases showing variable color intensities as shown in Fig. 9.

On comparing these results with our previously developed enhanced strip and that obtained after application of the same collected saliva samples on commercially available qualitative kit (Rightsign Kit-China) for detection of SARS-CoV-19 antigen in saliva samples, low sensitivity and specificity was observed on using the commercial kit (Table 4).

**Table 4:** Comparing Sensitivity and specificity of our developed LFTSs (BS-Nbs & Enhanced-Nbs strip) results with that of commercially available kit (Rightsign Kit REF ICOVG-C81 China) for rapid detection of SARS-CoV-2 antigen in saliva samples.

	Sensitivity (%)	Specificity (%)
Rightsign Kit	72.25	96.51
Enhanced strip	91.14	98.57
BS strip	95.21	99.29

### Application of the strip on health care worker groups:

Table 5 displayed the data of health care workers using BS-strip after 4 and 6 days. The specificity of technique was 100%, in both high risk and paramedic groups. By the 4th day, the sensitivity of the strip in high-risk group (88.9%) was higher than that of the paramedic group (66.7%). By increasing the time to the 6th day, the sensitivity was elevated, in both high risk (90.9%) and paramedic (80.0%) groups. According to the kappa test, in the high-risk group, the agreement level between the PCR and the strip by the 4th day (0.0.911) and at the 6th day (0.918) was almost perfect for both. However, in the paramedic group, the agreement level was (0.773 = moderate) at the 4th day while (0.857 = substantial) at the 6th day.

**Table 5:** Accuracy measures of biotin Streptavidin (BS) strip using PCR as a reference test in health care worker groups.

	Day	PCR (−) strip (−)	PCR (+) strip (+)	PPV (%)	NPV (%)	Accuracy (%)	Sensitivity (%)	Specificity (%)	kappa	Kappa SE	*P*-value
High risk group (n = 25)	4	16/17	9/8	100.00	94.10	96.0	88.9	100.00	0.911	0.087	0.000
	6	14/15	11/10	100.00	93.30	96.0	90.9	100.00	0.918	0.08	0.000
Paramedic group (n = 20)	4	17/18	3/2	100.00	94.40	95.0	66.7	100.00	0.773	0.216	0.000
	6	15/16	5/4	100.00	93.80	95.0	80.0	100.00	0.857	0.138	0.000

PPV, positive predictive value; NPV, negative predictive value.

*P* < 0.00 and *P* < 0.000: represent significant agreement.

## Discussion

SARS-CoV-2 antigen tests typically provide rapid results and are less expensive than RT-PCR, but they are generally less sensitive^11^. In order to identify the existence of a particular viral antigen, antigen tests for SARS-CoV-2 utilize immunoassays, which can be done through point-of-care, laboratory-based, or self-testing methods^12^. Due to their lower sensitivity, developing highly sensitive, rapid, and reliable techniques for COVID-19 diagnosis is a significant step towards early diagnosis and prevention of further infections, especially among healthcare workers (HCWs)^13^. In our previous work, we developed an enhanced signal strip for the detection of SARS-CoV-2 SI antigen in saliva from patients confirmed by RT-PCR, using gold-conjugated Nbs and ACE-2 as a matched pair of antigen probes^6^. This strip resulted in a sensitivity of 91.66% and specificity of 98.57% with a limit of viral detection (LVD) of 0.2 × 104 copies/ml. Therefore, we attempted to further improve the sensitivity and specificity by utilizing the biotin-streptavidin system. Streptavidin is widely used in molecular science due to its highly selective and stable interaction with biotin^14^.

We chose to implement the SARS-CoV-2 S1 antigen in our detection assay as it is a superior target compared to the nucleocapsid antigen. Previous studies have shown that the nucleocapsid antigen of SARS-CoV-2 can cross-react with other coronaviruses, which raised concerns about its reliability for seroprevalence studies^15^(34). The extensive use of nucleocapsid antigen for rapid COVID-19 detection has led to concerns from the CDC and FDA regarding the potential for false positive results in the community^16^(7). However, it has been found that the cross-reaction of pre-existing antibodies in SARS-CoV-2 negative individuals with the spike protein of endemic and seasonal coronaviruses is minimal and mainly directed against S2^17^(31). This supports the use of the S1 antigen, which allows for the specific binding of the ACE-2 peptidase domain to the SARS-CoV-2 S1 protein. By employing ACE-2 as the capturing antigen probe, we ensure strong binding specificity to the spike protein only.

Streptavidin is a tetrameric protein with a high binding affinity to biotin (in the low femtomolar range) and possesses four biotin binding sites^18^. These properties make the biotin-streptavidin assay a common choice in various biotechnological applications, including bioanalytical immunoassays^19^. Compared to other covalent and non-covalent bonds, the avidin–biotin system offers advantages such as efficient operation, amplification of weak signals, and the ability to use highly diluted primary antibodies^20,21^. The main advantage of this system is its high-affinity interaction, which remains stable against proteolytic enzymes, pH, temperature, and denaturing reagents^22^. As a result, the avidin–biotin interaction is a valuable tool in biomedical and nanotechnological applications^23^. Furthermore, this system is easy to synthesize and has minimal effects on biomolecule activity^24^. Moreover, using streptavidin–biotin system, based on the high binding affinity of streptavidin to biotin, is in favor of the employment of smaller amounts of Nbs. Moreover, the system requires the use of diluted ACE-2, and does not include a conjugate pad in the test design. All these factors together reduce the cost of biotin-streptavidin system when compared to our previously developed enhanced strip assay.

In this work, we found that the modified strip using the biotin-streptavidin system (BS) exhibited higher sensitivity (95.21%) and specificity (99.29%) compared to our previously developed enhanced signal strip (87.22% and 97.86% respectively). Moreover, the BS LFTS allowed a lower detection virus limit (LVD) of 0.2 × 104 copies/ml, surpassing the previously established enhanced signal strip LVD (0.4 × 104 copies/ml). These findings were attributed to the high-affinity interaction between biotin and streptavidin, facilitated by the four binding sites for biotin on streptavidin. Although sandwich ELISA is well-known and widely applied^25^, the assay approach utilizing the streptavidin–biotin interaction exhibited a tenfold higher sensitivity and reduced costs by 20-fold compared to RT-PCR^26^. We introduced several modifications for our previously developed enhanced signal strip, to enhance the sensitivity and specificity, while also reducing the cost of SARS-CoV-2 S1 antigen detection in saliva^6^. These modifications included bypassing several fabrication steps such as dispersing the conjugate pad, optimizing the strip, and employing a small sample volume (5 µl). Furthermore, the implementation of the BS system, allowed for reduced amounts of Nbs used and the application of diluted ACE-2. All these modifications contribute to cost reduction and decreased test performance time. The current biotin-streptavidin strips demonstrated stability for up to 4 months at room temperature without any changes in their reactivity.

Previous studies by Lakshmipriya et al. (2016) observed that the sensitivity of ELISA used for certain analyses can be improved by utilizing the powerful non-covalent interaction of biotin-streptavidin with its high affinity. They also stated that the biotin-streptavidin conjugation strategy is commonly employed in ELISA protocols to increase the limit of detection (LOD)^27^. In our modified biotin-streptavidin strip, streptavidin gold-conjugated nanoparticles were predominantly used as a detector due to their distinct red color in the presence of the antigen, resulting from colloidal Plasmon resonance phenomena. Additionally, the large surface area-to-volume ratio of gold nanoparticles, allows for the coating of hundreds of monoclonal antibodies (mAbs) on their surface, leading to signal enhancement and increased assay sensitivity. Kamel et al. (2019) succeeded in developing LFTS based on the employment of gold nanoparticles with sensitive detection for CSA in urine and serum samples of patients with active schistosomiasis^28^. In our study, the overall sensitivity and specificity of the BS strip for the detection of S1 antigen were higher in saliva samples compared to nasopharyngeal swabs. These findings were nearly the same as those in our previous study using ELISA^5^, thus avoiding the pain and discomfort associated with the nasopharyngeal swab process.

The sensitivity of NP swabs compared to saliva samples is a topic of debate. A research study conducted by Jung et al. in 2023 concluded that the sensitivity of detecting the S1 antigen in NP samples is higher than in saliva samples^29^. However, it's worth noting that they used RT-PCR for their testing. Conversely, other studies support our findings. For instance, in a study by Teo et al. in 2021, they discovered that saliva samples were both sensitive and viable for diagnosing COVID-19. In our work, we recommend using saliva samples instead of NP samples to avoid the harsh manipulation involved in collecting NP samples. Additionally, NP sample collection relies on thorough swabbing of the nasopharynx, which is done blindly and requires highly trained healthcare workers. This procedure could potentially account for the lower sensitivity of NP swabs, leading to an increased number of false-negative results^30^.

The COVID-19 pandemic is a healthcare crisis that has had an unprecedented impact on healthcare services, notable morbidity and mortality of the public and healthcare workers (HCWs), economic repercussions, and significant psychological effects^31,32^. Healthcare providers are the frontline soldiers battling against the coronavirus disease 2019 (COVID-19) pandemic. They are a highly susceptible subpopulation due to their time spent caring for COVID-19 patients^33^. In the clinical practice of healthcare workers, another important variable to consider is the exhaled air dispersion distance during oxygen administration and ventilatory support^34,35^.

Our BS strips showed high sensitivity when tested with samples from 45 health workers, and the Kappa coefficient of agreement between PCR and the BS strip was almost perfect, indicating the accuracy of the BS strip. This is a great advantage of our sensitive, rapid, non-invasive BS strip over other invasive techniques (e.g., PCR) or less accurate techniques (Ag detection strips), especially for healthcare workers who usually need regular check-ups as they are at continuous risk of infection.

## Conclusion

During the COVID-19 pandemic, early diagnosis was critical for containing disease spread. In this study, the LFTS was improved through the employment of the biotin-streptavidin system to increase the sensitivity and specificity of detecting SARS-CoV-2 S1 antigen. BS-based LFTS offers a more rapid, sensitive, and cheaper assay for the early diagnosis of COVID-19, which is of utmost importance for the rapid screening, prevention, and control of disease spreading among the population, especially healthcare workers.

## Data Availability

The datasets analyzed during the current study available from the corresponding author on reasonable request.
